# Is high-density lipoprotein cholesterol a prognostic marker in epithelial ovarian cancer?

**DOI:** 10.1590/1806-9282.20242079

**Published:** 2025-10-17

**Authors:** Mahmut Uçar, Mukaddes Yılmaz, Eda Erdiş, Birsen Yücel, Teoman Şakalar

**Affiliations:** 1 Sivas Cumhuriyet University, Department of Medical Oncology – Sivas, Turkey.; 2 Sivas Cumhuriyet University, Department of Radiation Oncology – Sivas, Turkey.; 3 Kahramanmaraş Sütçü İmam University, Department of Medical Oncology – Kahramanmaraş, Turkey.

**Keywords:** Epithelial ovarian cancer, Cholesterol, Survival, Prognosis

## Abstract

**OBJECTIVE:**

The aim of the study was to assess the prognostic potential of high-density lipoprotein cholesterol levels in predicting survival for patients with epithelial ovarian cancer.

**METHODS:**

This is a retrospective observational study. The cutoff value for high-density lipoprotein cholesterol was determined through receiver operating characteristic analysis, revealing a value of 45 mg/dL. Patients in Group I had high-density lipoprotein cholesterol values <45 mg/dL, while patients in Group II had high-density lipoprotein cholesterol values ≥45 mg/dL.

**RESULTS:**

A total of 219 patients participated in the study, including 78 (36%) in group I and 141 (64%) in group II. high-density lipoprotein cholesterol (HR 0.44, 95%CI 0.27–0.73, p=0.001), age ≥65 (HR 3.02, 95%CI 1.87–4.58, p<0.001), and stage (HR 3.68, 95%CI 1.07–12.67, p=0.038) were identified as independent prognostic factors for overall survival in the multivariate analysis. High-density lipoprotein cholesterol (HR 0.44, 95%CI 0.27–0.72, p=0.001) and N1b (HR 2.32, 95%CI 1.33–4.03, p=0.003) were independent prognostic factors for disease-free survival in the multivariate analysis.

**CONCLUSIONS:**

In epithelial ovarian cancer, high-density lipoprotein cholesterol levels were prognostic for both overall survival and disease-free survival.

## INTRODUCTION

Epithelial ovarian cancers remain the second leading cause of death among gynecological cancers^1^. Advanced-stage disease occurs in 75% of epithelial ovarian cancer cases. Although there have been advancements in treatment options, including major surgical procedures and novel adjuvant therapies, the overall survival (OS) rates remain at 40% for stage III and 20% for stage IV^2^.

The composition of high-density lipoprotein cholesterol (HDL-C) includes high-density lipoproteins (HDLs) and amphiphilic lipids, such as phospholipids, sphingolipids, and some free cholesterol located on the exterior. Hydrophobic lipids, specifically cholesteryl esters and triglycerides, form the primary core. The main protein present in HDL is Apolipoprotein A-I (apoA-I), which is vital for maintaining the stability of HDL's structure^3^. The role of HDLs is vital in reverse cholesterol transport, which may assist in removing cholesterol from malignant cells, thereby influencing their homeostatic regulation^4^. Moreover, HDL may affect various pathways, including apoptosis, angiogenesis, inflammation, oxidation, and immunomodulatory functions, all of which are significant in cancer biology. The protective role of HDLs in preventing oxidative modification of LDL (low-density lipoprotein) is attributed to their anti-oxidative properties, which may be augmented by HDL-associated proteins and enzymes, including apoAI, apoE2, apoAIV, apoJ, PON1, and LCAT^5–7^. In addition, HDL possesses the ability to suppress the secretion of proinflammatory cytokines while enhancing the expression of adhesion molecules on endothelial cells and leukocytes, thereby demonstrating anti-inflammatory properties^8^. The various functions of HDL often result in mechanisms that suppress tumorigenesis.

Maintaining membrane homeostasis and function in healthy cells heavily depends on lipids. In contrast, many diseases, such as metabolic disorders, immune-related issues, central nervous system disorders, and cancer, alter the activity of lipid metabolic enzymes and pathways^9^. Cancers of the endocrine system particularly necessitate increased cholesterol levels due to their heightened hormone and steroid production^10^. Nonetheless, studies have examined the links between triglycerides, LDL cholesterol, and HDL-C fluctuations in relation to gynecological, breast, lung, colon, and prostate cancers. The ongoing discussion revolves around whether these lipid alterations are a consequence of cancer or if they play a role in causing the disease^11^. Onwuka et al. showed that the HDL profile was lower in epithelial ovarian cancer patients than in healthy controls^12^. Conversely, Trabert et al. found no association between HDL and epithelial ovarian cancer risk^13^. Biases, such as study population, patient selection, and study design, may have influenced the results of these studies and could have led to differing outcomes. Moreover, it remains controversial whether lipid changes are a significant trigger for the development of epithelial ovarian cancer or whether they occur as a consequence of a person's impaired health status. There is a lack of studies examining the prognostic importance of HDL-C levels in individuals with epithelial ovarian cancer. Our research focused on determining whether HDL-C levels can reliably predict survival rates in these patients.

## METHODS

The study was designed as a retrospective observational study. It involved individuals admitted to the cancer department of two prominent research hospitals from January 01, 2010, to December 30, 2024. Information was sourced from the hospital's electronic information system for analysis. Approval number 2024/12-14 was granted to the study by the institutional ethical committee.

Patients diagnosed with epithelial ovarian cancer who had their HDL-C value measured at diagnosis, underwent standard treatments, and had complete follow-up data were included in the study. The study excluded patients with known autoimmune diseases, acute or chronic kidney disease, or other malignancies. The cut-off value for HDL-C was determined using ROC analysis. Participants were divided into two groups: the first group with low HDL-C (Group I) and the second group with high HDL-C (Group II). Demographic information and laboratory results were collected from the hospital's patient record system. Patients who underwent primary surgery received adjuvant chemotherapy based on platinum and taxanes. Those ineligible for primary surgery received 3–4 cycles of neoadjuvant chemotherapy. Interval cytoreduction was performed for eligible candidates, followed by additional cycles of chemotherapy in the adjuvant setting. If resectable disease was not present after neoadjuvant chemotherapy, primary medical treatment without surgery was recommended. Clinical staging was performed according to International Federation of Gynecology and Obstetrics (FIGO) staging. OS was defined as the time between the date of diagnosis and the date of last follow-up or exitus, and disease-free survival (DFS) was defined as the time between the date of diagnosis and the date of recurrence, the date of exitus, and the date of last follow-up for those who did not develop recurrence.

### Statistics

The statistical analysis was conducted using the Statistical Product and Service Solutions (SPSS) software, version 23, developed by IBM Corporation, located in Armonk, New York, USA. For the evaluation of non-categorical variables, the comparison of groups utilized Student's t-test in cases of normal distribution and the Mann-Whitney U test when the distribution was abnormal. To assess the relationships between categorical variables, the chi-square test was utilized. The survival times were analyzed using the Kaplan-Meier technique. Factors affecting survival were analyzed by univariate analysis using the log-rank test. The possible factors identified with univariate analyses were further entered into the Cox regression analysis, with backward selection, to determine independent predictors of survival. The p-value of <0.050 was considered statistically significant.

## RESULTS

A total of 219 patients were enrolled in the study. Among them, 78 (36%) patients were classified as having low HDL-C, also referred to as group I, while 141 (64%) patients were classified as having high HDL-C, referred to as group II. The median age was 56 (27–84). The median cancer antigen 125 (CA-125) value was 640 (6.6–12,691) U/mL.

In the receiver operating characteristic (ROC) analysis, the cut-off for the HDL-C with the best sensitivity and specificity was 45 mg/dL [area under the curve: 0.772 (0.676–0.867), p<0.004]. There was no significant difference in age, diabetes mellitus, body mass index, statin use, disease stage, or median CA-125. There was a statistically significant difference between the groups in terms of hypertension (p=0.046), and N stage (p=0.049). When the treatments of the patients were compared, primary surgery was performed more in group 2 (74 vs. 90%; p=0.002). The groups received adjuvant chemotherapy similarly. Recurrence was more frequent in group 1 (p=0.005). A comparison of demographic and clinical features, treatment procedures, and patterns of recurrence between the groups is presented in Table 1.

**Table 1:** Comparison of demographic and clinical features, treatment procedures, and patterns of recurrence of the groups.

		All patients n=219 (100)	Group I n=78 (36%)	Group II n=141 (64%)	p
Median age	Year (range)	56 (27–84)	61 (31–78)	55 (27–84)	0.059
Diabetes mellitus	No	148 (68)	51 (65)	97 (69)	0.356
	Yes	71 (32)	27 (35)	44 (31)	
Hypertension	No	88 (40)	25 (32)	63 (45)	0.046
	Yes	131 (60)	53 (68)	78 (55)	
Body mass index	Normal	65 (33)	20 (28)	45 (36)	0.491
	Overweight	59 (30)	24 (33)	35 (28)	
	Obese	74 (37)	28 (39)	46 (36)	
Statin use	No	146 (67)	47 (60)	99 (70)	0.089
	Yes	73 (33)	31 (40)	42 (30)	
Stage	Stage I–II	38 (17)	9 (11)	29 (21)	0.064
	Stage III–IV	181 (83)	69 (89)	112 (79)	
N stage	N0	112 (51)	36 (46)	76 (54)	0.049
	N1a	28 (13)	6 (8)	22 (16)	
	N1b	45 (21)	18 (23)	27 (19)	
	Nx	34 (15)	18 (23)	16 (11)	
CA-125	<Median	80 (49)	32 (56)	48 (45)	0.123
	≥Median	83 (51)	25 (44)	58 (55)	
Primary surgery	No	34 (15)	20 (26)	14 (10)	0.002
	Yes	185 (85)	58 (74)	127 (90)	
Interval debulking	No	165 (75)	63 (81)	102 (72)	0.110
	Yes	54 (25)	15 (19)	39 (28)	
Adjuvant chemotherapy	No	20 (9)	8 (10)	12 (8)	0.420
	Yes	199 (91)	70 (90)	129 (92)	
Recurrence	No	103 (47)	27 (35)	76 (54)	0.005
	Yes	116 (53)	51 (65)	65 (46)	
Recurrence pattern	Peritoneum	79 (68)	30 (59)	49 (75)	0.045
	Lymph node	63 (54)	27 (53)	36 (55)	0.470
	Solid organ	41 (35)	20 (39)	21 (32)	0.282

HDL-C: high-density lipoprotein-cholesterol; Group I: HDL-C <43 mg/dL; Group II: HDL-C ≥43 mg/dL; body mass index <25 normal, 25–29.9 overweight, ≥30 were obese; CA-125: cancer antigen 125; Median CA-125: 640 U/mL.

The 5-year OS (37 vs. 55%) and median OS (40 vs. 72 months) were better in group 2. Diabetes mellitus, hypertension, body mass index, statin use, and CA-125 value did not statistically affect survival in univariate analysis (p>0.050). At the same time, low HDL-C (p<0.001), age >65 years (p<0.001), advanced disease stage (p<0.001), and N stage, no primary surgery (p<0.001) were observed as factors negatively affecting OS. HDL-C (HR 0.44, 95%CI 0.27–0.73, p<0.001), age (HR 3.02, 95%CI 1.87–4.58, p<0.001), stage (HR 3.68, 95%CI 1.07–12.60, p=0.038), primary surgery (HR 0.43, 95%CI 0.21–0.87, p=0.020), N1a (HR 2.69, 95%CI 1.20–6.04, p=0.016) and Nx (HR 3.02, 95%CI 1.60–5.69, p=0.001) were independent prognostic factors for OS in multivariate analysis.

**Figure 1:**
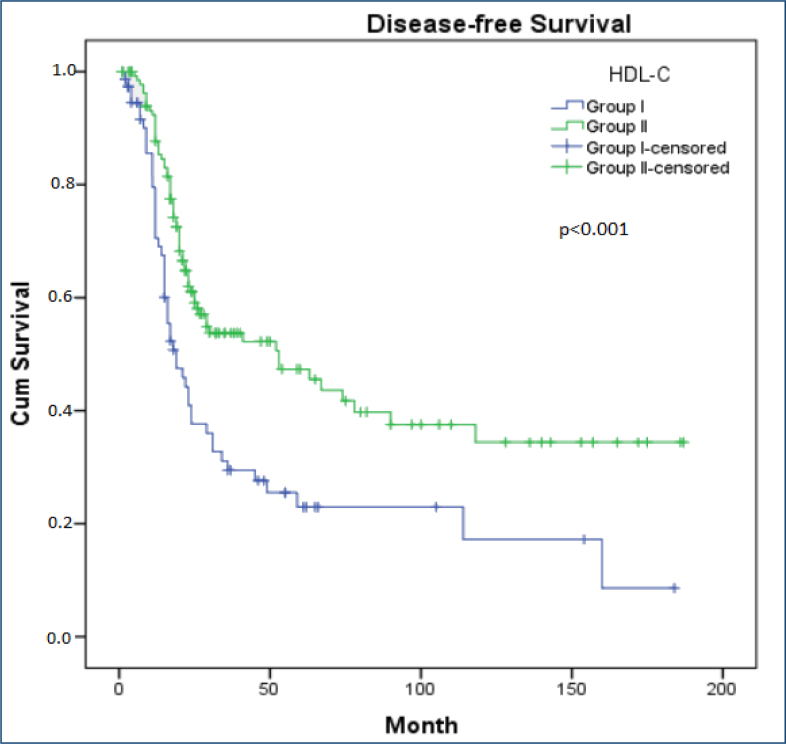
Kaplan-Meier curves for disease-free survival.

The 5-year DFS (23 vs. 48%) and median DFS (19 vs. 53 months) were better in group 2. Age, diabetes mellitus, hypertension, body mass index, and statin use did not statistically affect survival in univariate analysis (p>0.050). HDL-C (p<0.001), CA-125 value (p=0.015), stage (p<0.001), N stage (p=0.045), and primary surgery (p=0.003) were observed as factors affecting DFS. HDL-C (HR 0.44, 95%CI 0.27–0.72, p=0.001) was an independent prognostic factor for DFS in multivariate analysis. Table 2 shows the prognostic factors affecting OS and DFS. Figure 1 presents Kaplan-Meier curves for DFS.

**Table 2:** The prognostic factors for overall survival and disease-free survival in patients.

Survival time		Overall survival			Disease-free survival		
		The 5-year (%)	The median (month)	p-value	The 5-year (%)	The median (month)	p-value
HDL-C	Group I	37	40	<0.001	23	19	<0.001
	Group II	55	72		48	53	
Age	<65	57	72	<0.001	38	29	0.226
	≥65	23	30		36	24	
Diabetes mellitus	No	51	62	0.880	38	30	0.991
	Yes	43	57		37	26	
Hypertension	No	57	65	0.323	39	27	0.679
	Yes	43	55		38	29	
Body mass index	Normal	52	63	0.924	36	25	0.840
	Overweight	50	60		31	22	
	Obese	41	57		35	29	
Statine use	No	52	63	0.558	41	34	0.280
	Yes	43	57		32	26	
CA-125	<Median	55	68	0.090	51	67	0.015
	≥Median	37	49		22	23	
Stage	Stage I–II	80	180	<0.001	71	NR	<0.001
	Stage III–IV	41	49		30	23	
N stage	N0	66	124	<0.001	49	59	<0.001
	N1a	29	43		-	45	
	N1b	35	44		28	18	
	Nx	19	30		-	17	
Primary surgery	No	49	28	<0.001	-	16	<0.001
	Yes	55	70		42	36	
**Multivariate analysis**		**Overall survival**			**Disease-free survival**		
		**HR**	**95%CI**	**p**	**HR**	**95%CI**	**p**
HDL-C	Group I	1		<0.001	1		
	Group II	0.44	0.27–0.73		0.44	0.27–0.72	0.001
Age	<65	1		<0.001			
	≥65	3.02	1.87–4.58				
Stage	I–II	1			1		
	III–IV	3.68	1.07–12.6	0.038	2.31	0.92–5.81	0.073
N stage	N0	1			1		
	N1a	2.69	1.20–6.04	0.016	0.82	0.35–1.90	0.645
	N1b	1.73	0.98–3.08	0.059	2.32	1.33–4.03	0.003
	Nx	3.02	1.60–5.69	0.001	2.47	1.26–4.85	0.008
CA-125	<Median	1			1		
	≥Median	1.16	0.71–1.90	0.537	1.59	0.99–2.55	0.053
Primary surgery	No	RF			RF		
	Yes	0.43	0.21–0.87	0.020	0.77	0.40–1.47	0.437

HDL-C: high density lipoprotein-cholesterol; HR: hazard ratio; CI: confidence interval; CA-125: cancer antigen 125; Median CA-125: 640 U/mL; NR: not reached; RF: reference.

## DISCUSSION

The present study recognized HDL-C as a significant independent prognostic factor for both OS and DFS. Although the disease stages of the groups were similar, the higher incidence of early-stage disease in the high HDL-C group, along with more frequent primary surgeries performed in this group, was considered a confounding factor. Although contradictory, statin use—potentially a confounding factor based on literature regarding its relationship with cancer treatment outcomes—was comparable between the groups.

A meta-analysis discussing the diagnosis, prognosis, and prediction of lipomics highlighted the importance of lipid aberrations and lipid pathway alterations during the development of epithelial ovarian cancer^14^. Research indicates that key enzymes or proteins involved in cholesterol processing are linked to epithelial ovarian cancer, suggesting that disrupted cholesterol metabolism may contribute to the progression of the disease. According to Li et al., individuals with stages III–IV epithelial ovarian cancer showed lower levels of HDL-C compared to those with stages I and II cancer^15^. Similarly, Zhang et al. demonstrated the association between low HDL-C and a more advanced FIGO stage in patients diagnosed with epithelial ovarian cancer in a study^16^. Our research found that patients with low HDL-C exhibited numerically more advanced stages of the disease, but this was not statistically significant. However, the occurrence of N0 was more prevalent in patients with high HDL-C compared to those with low HDL-C. The small sample size may partly explain this unexpected result, and this finding could change with larger studies.

Cholesterol and its derivatives, known as oxysterols, act as both internal and external factors that promote tumor growth by altering the tumor microenvironment^17^. There is a lack of comprehensive studies that assess the prognostic role of HDL-C levels in patients diagnosed with epithelial ovarian cancer. In a retrospective analysis, Lin and colleagues demonstrated that lower levels of HDL-C adversely affected the outcomes for patients who underwent surgery for epithelial ovarian cancer^18^. Tang et al. demonstrated that in the advanced stages of epithelial ovarian cancer, a higher preoperative HDL-C level was associated with improved OS and progression-free survival^19^. In the study by Hongyan et al., they demonstrated that patients with significant serum HDL-C levels showed improved progression-free survival and OS when compared to their counterparts with lower HDL-C level concentrations^20^. In the present study, HDL-C was identified as a strong prognostic factor in epithelial ovarian cancer, alongside other well-known prognostic indicators such as age, stage, and CA-125. In other words, low HDL-C was associated with worse OS and DFS in patients with epithelial ovarian cancer. Furthermore, some critics might argue that individuals with low HDL-C and cancer often share numerous risk factors contributing to both conditions, including obesity, diabetes, hypertension, and environmental elements. When we evaluated the disease's prognosis alongside the factors affecting HDL-C levels, we found that comorbidities such as diabetes mellitus, obesity, and hypertension did not influence the prognosis in our cohort during the multivariate analysis. Moreover, HDL-C levels independently predict OS outcomes. In conclusion, HDL-C offers valuable insights into prognosis when considered alongside traditional laboratory assessments and clinical observations. Future studies should involve a larger sample size to validate the effectiveness of HDL-C in cases of epithelial ovarian cancer. These findings may illuminate the potential for using drugs that influence HDL-C metabolism in cancer treatment.

Our study has several limitations. Firstly, it is retrospective. HDL-C was measured, but it may not always accurately predict the quantity of HDL. Additionally, we did not measure quantity or function in our study. Nevertheless, this research is significant because few studies examine the prognostic efficacy of HDL-C in patients with epithelial ovarian cancer.

## CONCLUSION

HDL-C was found to be an independent prognostic factor for OS and DFS. However, there is a need for prospective studies with a large number of patients in which environmental factors such as dietary habits and patient treatments are balanced. If these results are confirmed, HDL-raising therapies can be pioneered.

## Data Availability

The datasets generated and/or analyzed during the current study are available from the corresponding author upon reasonable request.
